# A novel pathogenic variant of ARMC5 in a patient with primary bilateral macronodular adrenal hyperplasia: a case report

**DOI:** 10.1186/s12902-022-01128-x

**Published:** 2022-08-22

**Authors:** Wei Wang, Feng Wei

**Affiliations:** Department of Endocrinology, First Affiliated Hospital of Baotou Medical Collage, Inner Mongolia University of Science and Technology, Baotou, 014010 Inner Mongolia China

**Keywords:** Cushing’s syndrome, ACTH-independent macronodular adrenal hyperplasia, Primary bilateral macronodular adrenal hyperplasia, *ARMC5*, Variant, Case report

## Abstract

**Background:**

Primary bilateral macronodular adrenal hyperplasia (PBMAH), also known as adrenocorticotropic hormone (ACTH)-independent macronodular adrenal hyperplasia, is a rare cause of endogenous Cushing’s syndrome. In many familial cases of PBMAH, the variants in armadillo repeat containing 5 (*ARMC5*) gene are found to be associated with the disease. Here, we report a case of PBMAH harboring a novel frameshift variant in *ARMC5* gene, which has not been previously reported in the literature.

**Case presentation:**

A 67-year-old woman was referred due to the clinical features of Cushing’s syndrome. Radiological imaging and hormonal testing were carried out. The serum levels of cortisol were remarkably increased at late night and did not suppress even after 1 mg of dexamethasone administration, while the plasma levels of ACTH hormone were decreased significantly. The patient underwent unilateral left-sided laparoscopic adrenalectomy, and the diagnosis of PBMAH was substantiated by histopathological analysis. Moreover, the partial envelope was incomplete and the cell proliferation index was low. Specifically, inhibin α-subunit ( +), syn focal ( +), Ki-67 ~ 3% ( +), CgA (-) and CEA (-) were observed. DNA sequencing data revealed that a novel frameshift variant (c.363_373delGCCAGTGCGCC, p.Pro122Alafs*61) was identified in *ARMC5* gene. However, this variant was not detected in the daughter of the patient. The rest of the family members, including her sister, son and two brothers, were not consented for genetic testing.

**Conclusions:**

Early detection of *ARMC5* variant status and familial screening might have important clinical implications for the diagnosis and prognosis of PBMAH patients. A novel *ARMC5* frameshift variant (c.363_373delGCCAGTGCGCC, p.Pro122Alafs*61) was identified to be associated with the pathogenesis of PBMAH. *ARMC5* sequencing may improve the identification of a causative gene variant for PBMAH and allow earlier diagnosis of this disease.

## Background

Primary bilateral macronodular adrenal hyperplasia (PBMAH) is a rare type of Cushing syndrome (CS), accounting for less than 1% of all endogenous CS cases [1–3]. Generally, the disease progresses slowly over time. One possible explanation is that an inefficient steroidogenesis and a large adrenal mass can increase cortisol secretion, thereby leading to Cushing syndrome. PBMAH is characterized by enlarged bilateral adrenal glands, adrenal nodules larger than 10 mm in diameter (often close to 30 or 40 mm), and increased cortisol production. The bilateral appearance of nodules puts forward the hypothesis of potential germline genetic predisposition to the disease. Although most cases of PBMAH are apparently sporadic, there is sufficient evidence to prove the possibility of familial cases and indicate the underlying genetic component of this disease [3–6].

In recent years, it has been reported that the inactivating variants in *ARMC5* (Amadilo repeats containing 5; OMIM 615,549) gene located on chromosome 16p11.2 is the most common underlying genetic cause of PBMAH [7, 8]. Compared to those harboring wild-type *ARMC5*, PBMAH patients with *ARMC5* variants may have more advanced Cushing's syndrome. Specifically, the average age at diagnosis was lower in *ARMC5* variant group than in wild-type *ARMC5* group, while the prevalence of clinical CS and hypertension as well as the weight and number of adrenal nodules were higher [6]. The protein encoded by this gene has a presumed tumor suppressor function, and different types of variants in *ARMC5* are thought to be related to the pathogenesis of PBMAH [9]. These variants can cause the *ARMC5* protein to be shorter or inactive, leading to dedifferentiation of adrenal cortex cells and the growth of bilateral masses [10]. *ARMC5* variants were first discovered in sporadic PBMAH cases [4] as well as familial cases. The germline *ARMC5* variant in PBMAH cases can act as the first "hit" by inactivating the allele responsible for its protein expression, while subsequent somatic variants in the gene may lead to the development of adrenal nodules and cortisol overproduction [11–14]. In this study, we discovered a new type of frameshift *ARMC5* variant (c.363_373delGCCAGTGCGCC, p.Pro122Alafs*61) in patients with PBMAH, which has not been previously reported in the literature. This pathogenic variant can serve as a novel marker for PBMAH, which is of utmost significance for early diagnosis and genetic counseling.

## Case presentation

A 67-year-old Chinese woman was examined in our endocrinology department at the First Affiliated Hospital of Baotou Medical Collage, Inner Mongolia University of Science and Technology on September 23, 2019, because she had fatigue, edema, high blood pressure for 20 years and intermittent petechiae for 8 years. Physical examination showed that her blood pressure and pulse were 150/79 mmHg and 90 beats/min, respectively, along with typical CS features such as full moon face, reddened cheeks, central obesity, thin limbs, atrophic skin, reddish-purple striae, ecchymosis, petechiae, and lower extremity edema (Table 1). However, no obvious fat pad was formed on the back of the neck and supraclavicular fossa, and no purple streaks appeared on the bilateral underarms, abdomen and back. Oral glucose tolerance test was carried out for glucose metabolism assessment. The fasting blood glucose level of this patient was 9.8 mmol/L, and the blood glucose level two hours after taking sugar was 26.2 mmol/L, which met the WHO diagnostic criteria for diabetes. Laboratory examination after admission indicated that the blood level of potassium was 3.46 mmol/L (normal range: 3.50–5.50 mmol/L), while the urinary level of potassium was 32.80 mmol/24 h (normal range: < 25 mmol/24 h). These findings suggest that potassium ions may be lost from the body through the renal pathway.

**Table 1:** The main comorbidities at diagnosis and their management

	Diagnosis	Treatment (Before surgery)	Treatment (After surgery)	Follow up
Glucose metabolism assessment	Diabetes	Insulin + 2 oral hypoglycemic drugs	Stopped insulin and received two oral hypoglycemic drugs	1 Hypoglycemic drug
Blood pressure	Hypertension	3 Antihypertensive drugs Blood pressure = 145/95 mmHg	2 Antihypertensive drugs Blood pressure = 140/90 mmHg	1 antihypertensive drug Blood pressure = 130/80 mmHg
Blood lipid metabolism	Hyperlipidemia	Lipid-lowering drugs	Lipid-lowering drugs	Deactivate
Bone metabolism	Bone loss	Calcium + Vitamin D	Calcium + Vitamin D	Calcium + Vitamin D
Sleep	Sleep disorder	Diazepam	Discontinue the drug	Discontinue the drug
Skin	Scattered ecchymosis	Scattered ecchymosis	Scattered ecchymosis	No ecchymosis

Considering the association between potassium loss and the renal pathway, the adrenal function was evaluated through RAAS system (standing position). The levels of renin, angiotensin II, aldosterone, cortisol and ACTH are presented in Table 2. At the same time, the levels of aldosterone and renin were determined by an automated chemiluminescence immunoassay. According to the guideline [15], the values of aldosterone (ng/dL), renin (ng/L) and aldosterone/renin ratio were calculated as 3.8, 5.7 and 7.7, respectively. These results were considered to be negative for primary aldosteronism. Blood and urine catecholamine hormones are within the normal range (Table 2). Upon admission, the serum levels of cortisol at 8:00 and 24:00 were 26.40 µg/dL (normal range: 4.26–24.85 µg/dL) and 16.69 µg/dL (normal range: 2.5–15.8 µg/dL), respectively; while the 24-h urine free cortisol (2 times) levels ranged from 48.40 to 107.15 nmol/24 h (normal range: 4.20–35.50 nmol/24 h). In contrast, the plasma ACTH levels were decreased at both time points, with 3.03 ng/L (normal range: 7.2–63.3 ng/L) and 1.34 ng/L, respectively. Furthermore, the overnight dexamethasone suppression tests indicated that blood cortisol level (23.53 µg/dL, normal range: 2.5–15.8 µg/dL) was not suppressed after taking 1 mg of dexamethasone (Table 2).

**Table 2:** List of laboratory test results

**Laboratory items**	**Normal range**	**Measured value**
Potassium	3.50–5.50 mmol/L	3.46 mmol/L
Urinary potassium level	< 25 mmol/24 h	32.80 mmol/24 h
Cortisol (upright) (8:00, 2 times)	4.26–24.85 µg/dL	26.40–29.75 µg/dL
ACTH (upright)	7.2–63.3 ng/L	3.03 ng/L
Renin (upright)	4–38 ng/L	45.16 ng/L
Angiotensin II	49–252 ng/L	474.80 ng/L
Aldosterone	40–310 ng/L	162.34 ng/L
Aldosterone/renin ratio	3.8, 5.7 and 7.7	0.6
Cortisol (24:00)	2.5–15.8 µg/dL	16.69 µg/dL
h urine free cortisol (2 times)	4.20–35.50 nmol/24 h	48.40–107.15 nmol/24 h
Overnight dexamethasone suppression tests Cortisol (8:00)	< 1.8 µg/dL	23.53 µg/dL
ACTH (24:00)	7.2–63.3 ng/L	1.34 ng/L
Plasma adrenaline level	0–100 ng/L	43.16 ng/L
Plasma norepinephrine level	0–600 ng/L	154.83 ng/L
Plasma catecholamine level	0–100 ng/L	57.59 ng/L
Urine adrenaline level	0–20 µg/day	0.79 µg/day
Urine norepinephrine level	0–90 µg/day	11.12 µg/day
Urine catecholamine level	0–600 µg/day	16.45 µg/day

**Fig. 1:**
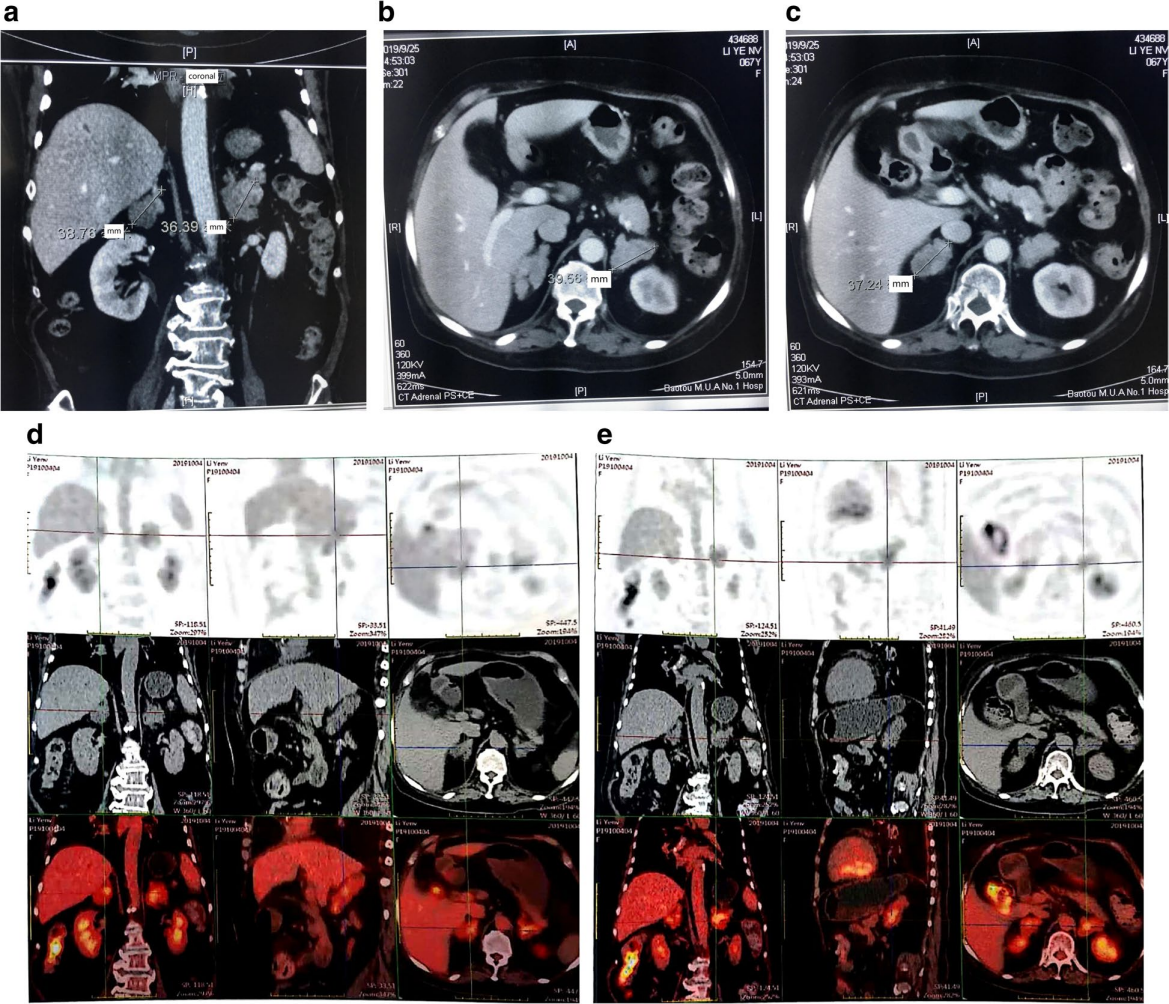
(**a**, **b**, **c**, **d** and **e**) **a**: Bilateral adrenal glands increased in size, with multiple nodular low-density shadows irregularly protruding, causing bilateral adrenal glands to appear "ginger-like" and enhanced with mildly uneven enhancement. Diagnostic considerations: bilateral nodular hyperplasia **b**: The longest diameter of the left adrenal gland is about 40 mm, with clear boundaries **C**: The longest diameter of the right adrenal gland is about 37 mm, with clear boundaries **d**: Increased volume of the left adrenal gland, with high metabolic heterogeneity **e**: Increased volume of the right adrenal gland, with high metabolic heterogeneity

For the imaging tests, adrenal gland computed tomography (CT) scan demonstrated that the bilateral adrenal glands were swollen, with multiple prominent nodules of varying sizes, soft tissue density and uniform density. The sizes of the left and right bilateral adrenal masses were 3.2 × 2.4 cm and 2.2 × 2.1 cm, respectively (Fig. 1a-c). By taking into account that bilateral adrenal lesions need to be differentiated from lymphoma and adrenal metastasis, fluorine-18-fluorodeoxyglucose positron emission tomography (18F-FDG PET)/CT scan was performed on this patient. The results showed that the volume of the bilateral adrenal glands was increased significantly with increasing metabolic heterogeneity, and the metabolism was improved further after delayed imaging (Fig. 1d and e). Bone density scan (dual-energy x-ray absorptiometry) demonstrated the abnormal values of Z-scores in the left hip (0.3) and lumbar spine (1.0), suggesting that bone loss is accelerated. Thoracic and lumbar spine lateral X-ray film indicated a lumbar spine scoliosis as well as L4-L5 and L5-S1 intervertebral space narrowing. For thyroid function evaluation, the values for TSH, FT3 and FT4 were 0.82 mIU/L (normal range: 0.35–5.5 mIU/L), 2.46 ng/L (normal range: 2.3–4.2 ng/L) and 10.70 pmol/L (normal range: 7.5–17.4 pmol/L), respectively. By combining the patient's medical history, clinical symptoms, physical examination and laboratory data, the diagnosis was confirmed to be PBMAH, which represents a rare cause of CS.

**Fig. 2:**
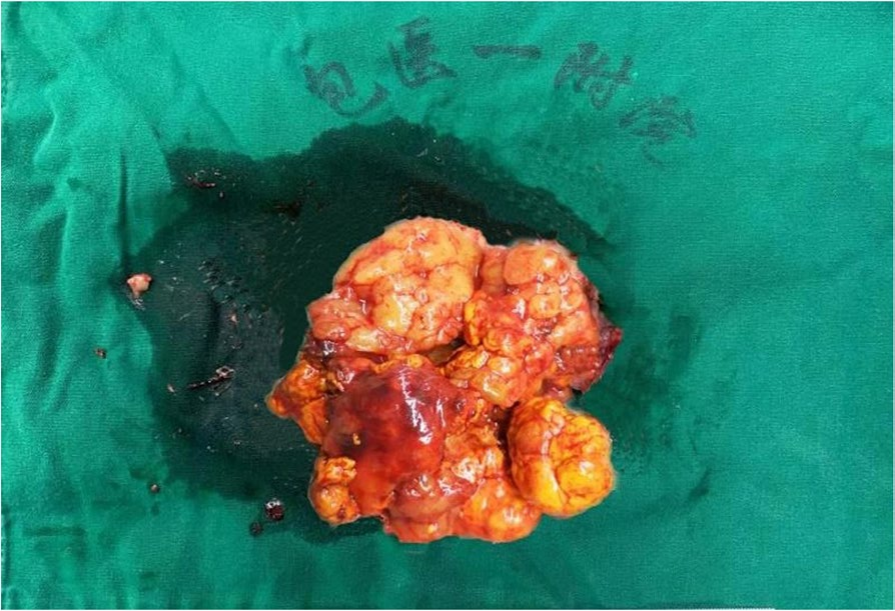
Visual observation: gray-yellow crushed tissue on the left side of the adrenal gland 4 cm*5 cm*2 cm, the surface seems to have a thin film, the cut surface is golden yellow

**Fig. 3:**
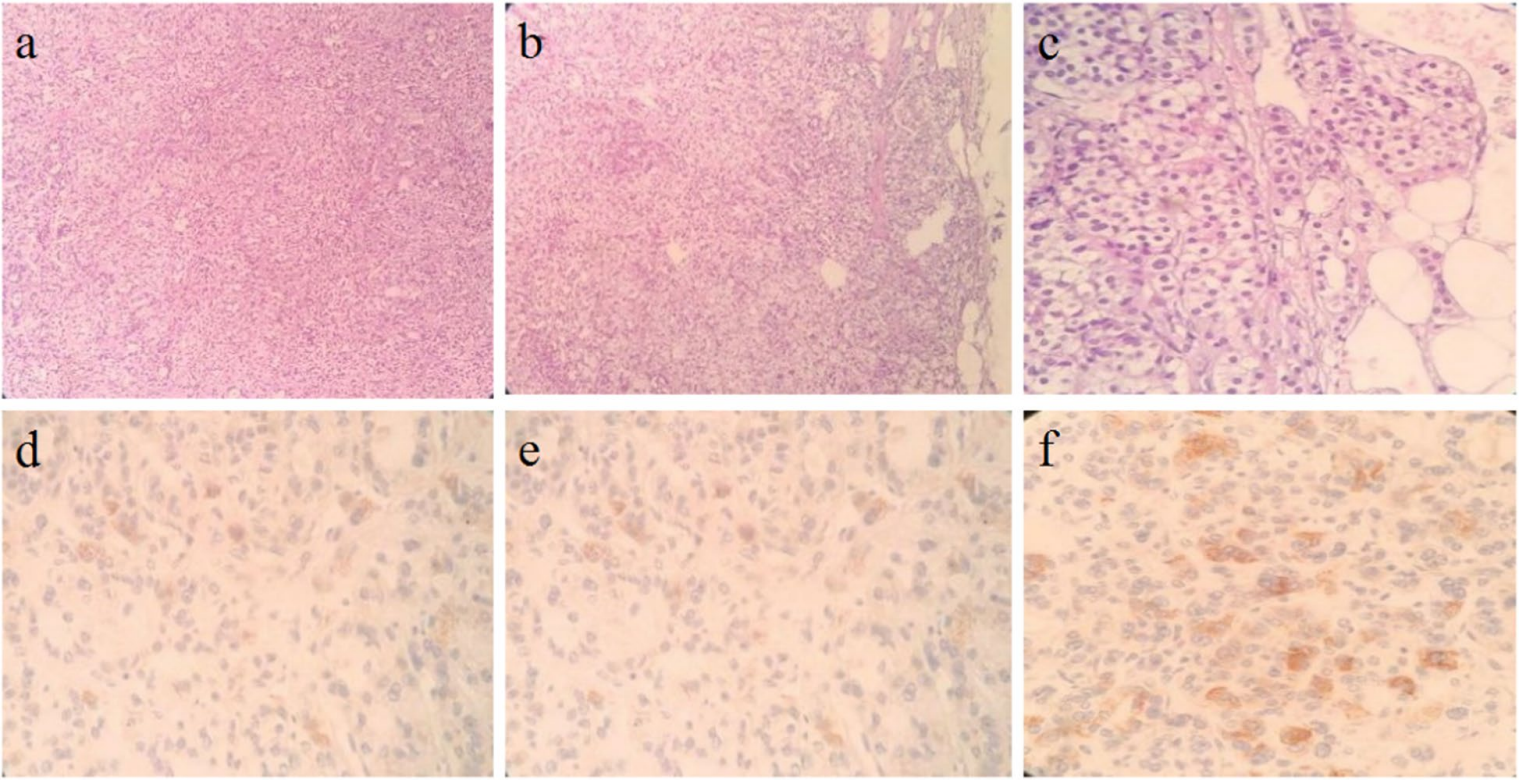
Pathology and immunohistochemistry findings Equipment parameters: Microscope: OLYMPUS BX53 Camera: C3CMOS10000KPA P/N: CP110000A Acquisition software: Image View Eyepieces 10x (**a**) The left adrenal gland tissue meets the characteristics of sebaceous adenoma (HE × 100). (**b**) Part of the capsule is incomplete (HE × 100). (**c**) Hyperplastic mass protrudes into the surrounding fat tissue (HE × 400). (**d**) Inhibin α-subunit ( +) (HE × 400). (**e**) Syn focal ( +). (f) Ki-67 of ~ 3% ( +) (HE × 400)

**Fig. 4:**
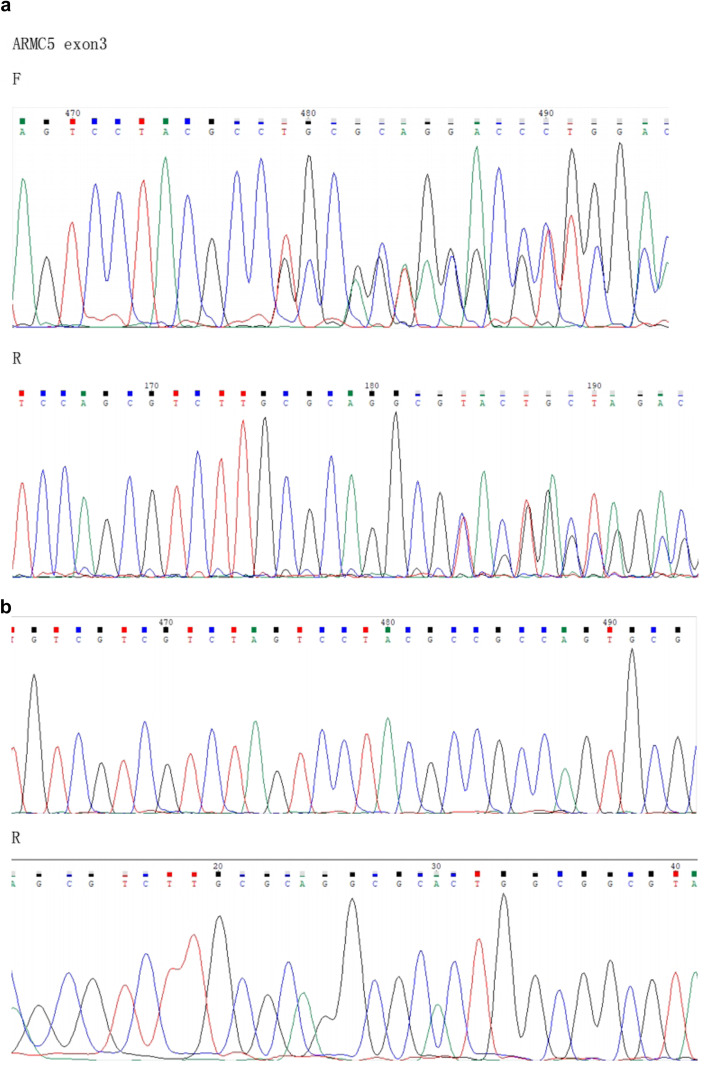
Genetic test results. This gene was detected by the NGS laboratory of Shanghai Institute of Endocrine and Metabolic Diseases (Department of Endocrinology and Metabolism, Ruijin Hospital, Shanghai Jiaotong University). **a**: Subject: There is a c.363_373delGCCAGTGCGCC variant in exon 16 of the *ARMC5* gene, namely, p.Pro122Alafs*61 **b**: Subject's first-degree relatives (daughter): The mutation was not detected

On October 20, 2019, the patient underwent left-sided adrenalectomy using laparoscopic retroperitoneal approach. Under laparoscopic separation, the dorsal side of the left upper adrenal pole to the diaphragm was first separated, and the ventral side of the left upper adrenal pole was then separated. The left adrenal mass was found, of which the size was about 6 cm × 5 cm, and it was nodular. Afterwards, the mass was completely removed, and electrocoagulation was used to stop bleeding. It was observed that the left adrenal gland lost its normal shape and exhibited a golden-yellow irregular nodular hyperplasia. The surgically removed adrenal gland showed that macronodular adrenal hyperplasia tended to have abundant compact cells without infarction. Pathological examination indicated that the grayish yellow crushed tissue on the left side of the adrenal gland was 4 cm × 5 cm × 2 cm, with a cut surface of golden-yellow in color (Fig. 2). Based on the light microscopic observation, adrenal adenoma was detected on the left adrenal gland, part of the area was incomplete, and a hyperplastic mass was protruded into the surrounding fatty tissue (Fig. 3a-c). Immunohistochemical analysis revealed that the left adrenal gland tissue was in line with the characteristics of adrenal adenoma, partly surrounded by a fibrous pseudocapsule, and the cell proliferation index was low. Among them, inhibin α-subunit ( +), syn focal ( +), Ki-67 ~ 3% ( +), CgA (-), CEA (-), Melan-A (-) and S-100 (-) were observed (Fig. 3d-f). After obtaining the written informed consents from the patient and her daughter, DNA was extracted from peripheral blood leukocytes. All coding and flanking intron sequences in *ARMC5* gene was amplified using Polymerase Chain Reaction (PCR) assay. PCR products were then subjected to DNA sequencing. For the variation nomenclature, the most common isoform (NG_034258.1 NM_001105247.2) was referred. After searching against ClinVar, Exome Aggregation Consortium (ExAC), 1000 Genomes, dbSNP database and UCSC Genome browser, a novel c.363_373delGCCAGTGCGCC variant was identified in *ARMC5* gene, which was predicted to be a frameshift variant (p.Pro122Alafs*61). This variant has not been previously reported in the literature. After screening the daughter of the patient, no similar *ARMC5* variant was detected (Fig. 4a and b).

This patient was followed up for a period of 3 months. On day 3 after surgery, the blood cortisol levels were 18.17 µg/dL (24:00 h) (normal range: 2.5–15.8 µg/dL) and 20.86 µg/dL (8:00 h) (normal range: 4.26–24.85 µg/dL), while the 24-h urinary free cortisol level was 66.29 nmol/24 h (normal range: 4.20–35.50 nmol/24 h). On day 5 after surgery, the re-measured blood cortisol levels were 17.58 µg/dL (24:00 h) (normal range: 2.5–15.8 µg/dL) and 19.37 µg/dL (8:00 h) (normal range: 4.26–24.85 µg/dL) while the 24-h urinary free cortisol level was 51.71 nmol/24 h (normal range: 4.20–35.50 nmol/24 h). On day 40 after surgery, the re-examination of cortisol levels showed remarkable declines in the blood (12.59 µg/dL at 24:00 h (normal range: 2.5–15.8 µg/dL) and 11.46 µg/dL at 8:00 h) (normal range: 4.26–24.85 µg/dL) and urine (27.11 nmol/24 h) (normal range: 4.20–35.50 nmol/24 h) samples, while a normal blood potassium level was obtained. On day 90 after surgery, fatigue was significantly improved, petechiae on the upper extremities were subsided, and the lower extremities were not swollen. For diabetes treatment, insulin therapy was stopped and 0.5 g oral metformin was given once daily. The blood glucose levels during fasting and two hours after a meal were approximately 7 and 10 mmol/L, respectively. For hypertension treatment, doxazosin tablets were discontinued, nifedipine controlled-release tablets (30 mg) were reduced from twice to once daily, and the blood pressure was regulated at approximately 130/85 mmHg. Significant improvement was observed for sleep disturbance. The reporting of this study conforms to CARE guidelines [16]. Ethics approval was not required for this case study.

## Discussion and conclusions

In this study, we reported a rare case of PBMAH who presented with typical PBMAH, PBMAH-associated CS and hypertension. PBMAH is a heterogeneous disease with varying degrees of hypercortisolism (from subclinical to overt CS), and the patients are often diagnosed at 50 to 60 years of age. Although hypercortisolemia is relatively mild, PBMAH often manifests with severe metabolic disturbances (e.g., hypokalemia). This suggests that particular attention should be paid to metabolic complications and radiological aspects (from massive nodular hyperplasia to macronodular adrenal hyperplasia) when performing the functional diagnosis of PBMAH patients [17]. Moreover, an early diagnosis can be difficult, as the clinical manifestations are asymptomatic and only becomes apparent with large nodules. In recent years, some scholars have shown that the incidence of PBMAH exhibits a certain familial aggregation, suggesting that genetic factors may play a key role in the pathogenesis of PBMAH [13, 18]. Variations in genes encoding melanocortin type 2 receptor and G protein-coupled receptor proteins can serve as the molecular mechanisms that contribute to intra-adrenal ACTH, but they are not the common causes [5, 8, 9]. In intra-adrenal ACTH patients, blood ACTH is usually suppressed, and its concentrations range from a low normal value to undetectable; however, their blood cortisol concentration is always higher than normal. Some studies have explained that adrenal chromaffin cells can synthesize and secrete ACTH under normal physiological conditions, while the non-chromosteroid-producing cells generated under pathological conditions can induce an excessive secretion of ACTH in the adrenal glands, thereby stimulating cortisol production and causing bilateral nodular hyperplasia [11].

Since the first discovery of *ARMC5* variants causing PBMAH, great efforts have been made to establish the importance of *ARMC5* protein in regulating adrenocortical homeostasis. Previous research has shown that PBMAH can become clinically apparent only with a second somatic variant in addition to *ARMC5*, as predicted by Knudson’s two-hit model [4]. PBMAH patient may carry a first germline variant and a second somatic variant in *ARMC5* gene, resulting in a loss of protein function and subsequent tumor formation. However, there is still a need for better understanding of *ARMC5* protein partners and signaling regulation that contribute to PBMAH. More than 40 frameshift variants, 30 missense variants, 27 nonsense variants and loss of heterozygosity have been described, supporting the possible roles of these genetic alterations during the inactivation or overexpression of *ARMC5* protein. As the molecular function of *ARMC5* is still unclear, it remains difficult to determine the deleterious potential of missense variants identified in the germline cells of patients with PBMAH. Most of the genetic association studies have focused on the missense variants found in less than 1% of the general population and in-silico predicted to be damaging using bioinformatics software programs. An important consideration is that even if there is a significant association between *ARMC5* variants and PBMAH pathogenesis, the resulting phenotypes may be varied due to the nature of the variants. It has been reported that *ARMC5* defects are associated with more severe PBMAH and CS, such as higher cortisol levels, larger adrenal glands and higher numbers of nodules, and *ARMC5*-mutated patients tend to have hypertension more often than wild-type patients [4, 11, 19]. In this case, *ARMC5* p.Pro121Alafs*61 variant was associated with the increased and decreased levels of cortisol and ACTH, respectively. However, these findings need to be verified in a larger cohort study.

Over the past decade, numerous pathogenic *ARMC5* variants have been identified in patients with PBMAH. Yu et al. [3] revealed eight new *ARMC5* variants, such as c.318delG (p.Ser107Argfs*30), c.523delG (p.Ala175Profs*7), c.622-623insC (p.Gln208Profs*15), c.1214delG (p.Gly405Alafs*56), c.1855C > T (p.Arg619*), c.2189C > A (p.Ser730*), c.2564delT (p.Val855Glyfs*62) and c.2599G > T (p.Glu867*), in 23 patients with sporadic PBMAH. Another study [13] has demonstrated that somatic (p.R502fs) and germline frameshift (c.323_324insC, p.A110fs*9) *ARMC5* variants are detected in PBMAH patients with intracranial meningioma. Liu et al. [20] identified a germline *ARMC5* variant (c.517C > T, p.Arg173*) in a sporadic PBMAH case with hyperplasia, CS and hypertension. More recently, Zhang et al. [21] found that a new *ARMC5* germline variant (c.52C > T, p.Gln18*) could be involved in the development of PBMAH. In the present study, our sequencing data also revealed a new frameshift variant (c.363_373delGCCAGTGCGCC, p.Pro121Alafs*61) in *ARMC5* gene. Further bioinformatics analysis indicated that p.Pro121Alafs*61 was indeed novel and had not been reported previously. After searching against ClinVar, Exome Aggregation Consortium (ExAC), 1000 Genomes, dbSNP database and UCSC Genome browser, p.Pro121Alafs*61 was classified as frameshift variant and had not been reported previously, indicating that this variant is indeed novel. Our results showed that this variant was associated with PBMAH case with intermittent petechiae, hypertension and diabetes as well as high cortisol and low ACTH levels.

In this report, we identified a novel *ARMC5* frameshift variant (c.363_373delGCCAGTGCGC, p.Pro121Alafs*61) in a patient with PBMAH, which could contribute to a better understanding of the pathogenesis of this disease. Our study extends the existing knowledge regarding the relationship between *ARMC5* and PBMAH pathogenesis. Early detection of *ARMC5* variant status and familial screening can allow for the effective treatment and prevention of this disease.

## Data Availability

The data that support the findings of this study are available from the corresponding author upon reasonable request.The *ARMC5* sequence is available in the GenBank database under accession number ON684457.

## Abbreviations

PBMAH: Primary bilateral macronodular adrenal hyperplasia
ACTH: Adrenocorticotropic hormone
CS: Cushing’s syndrome
ARMC5: Armadillo repeat containing 5
CT: Computerized tomography
PET: Fluorodeoxyglucose 18F-positron emission tomography
